# Obstructive Jaundice Due to Hilar Bile Duct Compression with Encasement of the Right Hepatic Artery

**DOI:** 10.4021/gr2009.10.1319

**Published:** 2009-09-20

**Authors:** Ryusuke Ito, Yuji Ishii, Tadashi Uwagawa, Shigeki Wakiyama, Hiroaki Shiba, Takeyuki Misawa, Yuichi Ishida, Hiroshi Kakutani, Shunichi Sadaoka, Katsuhiko Yanaga

**Affiliations:** a Department of Surgery, Jikei University School of Medicine, Tokyo, Japan; b Department of Endoscopy, Jikei University School of Medicine, Tokyo, Japan; c Department of Radiology, Jikei University School of Medicine, Tokyo, Japan

**Keywords:** Obstructive jaundice, Right hepatic artery, Hepatic cyst, Hilar bile duct, Cholangioscopy

## Abstract

A 68-year-old woman had a solitary 12.0 cm hepatic cyst with a septum. The cyst was located near the hepatic hilum and she presented with obstructive jaundice caused by compression of the hilar bile duct. Stenosis of the common hepatic duct was detected at the porta hepatis on endoscopic retrograde cholangiography (ERC), and encasement of the right hepatic artery at the same level was revealed by abdominal angiography. Transpapillary cholangioscopy showed compression and mucosal erosions of the hilar bile duct. After transpapillary cholangioscopy, the hepatic cyst became infected, for which emergency percutaneous transhepatic drainage was performed. As a result, the patient’s obstructive jaundice subsided. The mucosal erosions of the bile duct that existed at a site corresponding to the encasement of the right hepatic artery also improved. In conclusion, bile duct stenosis was considered to be caused by compression due to the hepatic cyst and the right hepatic artery.

## Introduction

Benign, non-parasitic liver cysts are usually asymptomatic and are found incidentally by abdominal ultrasonography or computed tomography (CT). Although such cysts occasionally cause complications, obstructive jaundice due to non-parasitic hepatic cysts is a rare event [1-5]. In contrast, anatomical variations of the biliary tree and surrounding structures are relatively common. We herein report a rare case of obstructive jaundice accompanied by changes of the bile duct due to compression by a large hepatic cyst and the right hepatic artery.

## Case Report

The patient was a 68-year-old woman with a history of hypertension and hyperlipidemia. She was found to have obstructive jaundice at a medical check-up in October 2005, and was referred to our hospital for further evaluation.

The patient was asymptomatic and her abdomen was soft. However, a firm mass was palpable at 5 finger-breadths below the right costal margin. The serum total bilirubin level was 4.0 mg/dL. Other abnormal laboratory data included the following: direct bilirubin, 2.7 mg/dL (normal range: 0.0 to 0.3); aspartate aminotransferase, 152 IU/L (normal range: 10 to 33); alanine aminotransferase, 105 IU/L (normal range: 6 to 35); alkaline phosphatase, 1,122 IU/L (normal range: 96 to 300); γ-glutamyl transpeptidase, 307 IU/L (normal range: 9 to 27); amylase, 189 IU/L (normal range: 30 to 130); carcinoembryonic antigen, 6.9 ng/ml (normal range: ≤ 5.8); CA19-9, 102 U/ml (normal range: ≤ 37); and DUPAN-II, 43 x 10^4^ U/ml (normal range: ≤ 150). She had a traveling history to Rebun island near Hokkaido in Japan three years ago, cystic echinococcosis is not rare.

**Figure 1:**
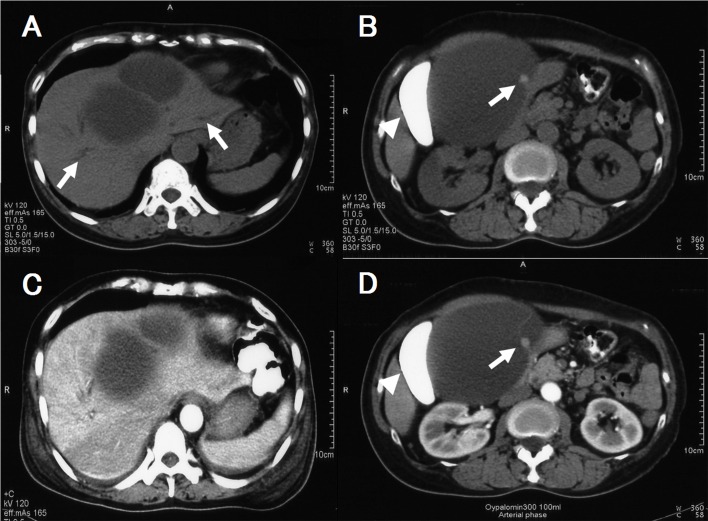
A and B: Plain CT after drip infusion CT cholangiography (DIC-CT), C and D: Enhanced CT after DIC-CT. A and C showed a hepatic cyst with septum near the hepatic hilum and slight dilatation of the intrahepatic duct (arrow). B and D showed a mural module which did not enhance with contrast material (arrow) as well as deformation of the gallbladder (arrowhead).

Ultrasonography (US) and computed tomography (CT) showed a hepatic cyst that measured 12.0 cm in diameter located near the porta hepatis (segment IV), as well as slightly dilated left and right intrahepatic bile ducts (Fig. 1A, 1B). US showed a mural nodule and a septum within the cyst. CT also detected a mural nodule that did not show contrast enhancement (Fig. 1C, 1D). The imaging findings combined with her traveling history suggested a diagnosis of cystic echinococcosis, but echinococus serology was negative.

**Figure 2:**
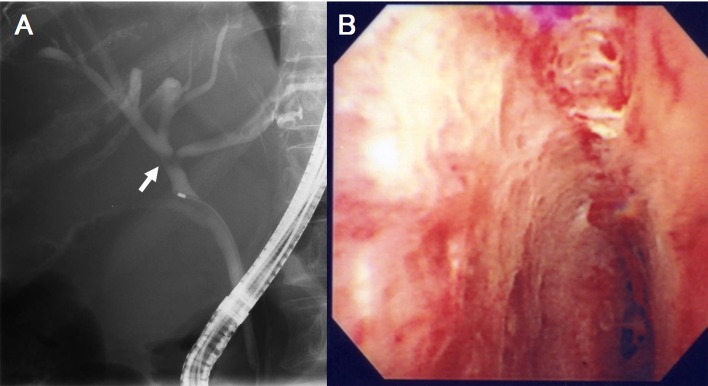
A: Endoscopic retrograde cholangiography (ERC) demonstrated a band-like filling defect of the hilar bile duct (arrow). B: Choledochoscopy demonstrated compression and mucosal erosive changes of the hilar bile duct.

**Figure 3:**
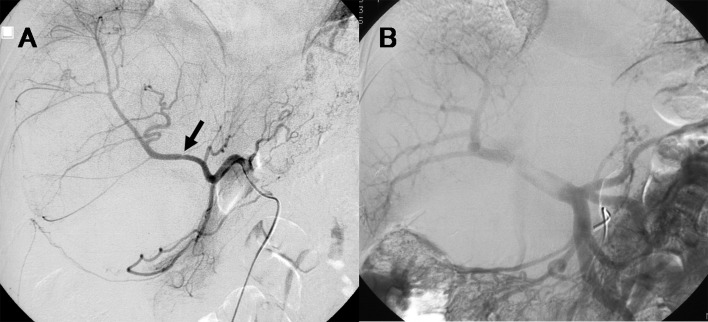
A: Abdominal angiography revealed distortion of the cystic and gastroduodenal arteries as well as slight encasement of the right hepatic artery (arrow). B: Mechanical displacement of the portal vein was observed, and the left branch of the portal vein was not visualized.

Endoscopic retrograde cholangiography (ERC) demonstrated a band-like filling defect of the common hepatic duct (Fig. 2A). Transpapillary cholangioscopy and endoscopic biopsy were also performed. Cholangioscopy showed compression and mucosal erosions of the hilar bile duct (Fig. 2B), while pathological examination did not identify any malignant cells. Abdominal angiography revealed distortion of the cystic and gastroduodenal arteries, as well as slight encasement of the right hepatic artery (Fig. 3A). Displacement of the portal vein was also observed, and the left branch of the portal vein was not visualized (Fig. 3B).

After transpapillary choledochoscopy, the patient developed acute pancreatitis and cholangitis. Papillary edema also occurred and her obstructive jaundice became worse, for which endoscopic retrograde biliary drainage (ERBD) was performed. Several days later, the patient developed right upper quadrant pain and fever. Abdominal US revealed a sludge echo inside the hepatic cyst, so a diagnosis of infected hepatic cyst was entertained. Subsequently, percutaneous transhepatic drainage of the cyst yielded milky yellowish fluid.

**Figure 4:**
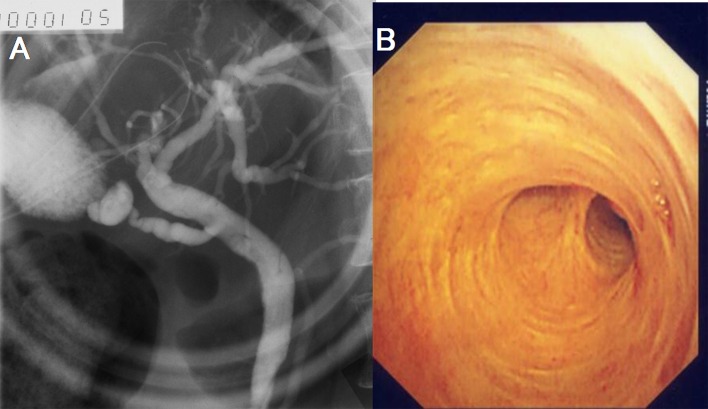
A: The filling defect of the bile duct disappeared on ERCP. B: Choledochoscopicexamination revealed normal bile duct without stenosis nor mucosal erosive changes.

Cystography showed no leakage of contrast or communication with the biliary tract. Bacterial and fungal cultures of the cyst contents detected α-streptococcus, while repeated cytologic examination was negative for malignant cells. The CA19-9 level in the cyst fluid was 12,240 U/ml, while the total bilirubin and amylase levels were 1.4 mg/dl and 46 IU/L, respectively. After percutaneous drainage, her fever subsided and her jaundice improved. The drainage tube was removed 40 days after placement. Although the DUPAN-II level also decreased, it was still 1 x 10^4^ U/ml as of 10 months after drainage and did not reach the normal range. The filling defect of the bile duct was no longer detected on ERCP (Fig. 4A), while choledochoscopic examination revealed a normal bile duct without stenosis or mucosal erosive changes (Fig. 4B). As of 18 months after percutaneous drainage, she remains well without jaundice or recurrence of the liver cyst.

## Discussion

A solitary non-parasitic hepatic cyst is rarely associated with obstructive jaundice [1-5]. We experienced a rare case of obstructive jaundice accompanied by stenosis and mucosal erosions of the bile duct due to compression by a hepatic cyst and the right hepatic artery. Before making a diagnosis of hepatic cyst, it is important to rule out cystadenoma or cystadenocarcinoma. In the present patient, imaging studies showed a mural nodule and septum in the cyst, but no enhancement was observed on contrast CT scans. Serological studies were negative, although echinococcosis was suspected from the imaging findings and her travel history. After transpapillary cholangioscopy, the cyst became infected and her jaundice was worsened.

A solitary non-parasitic hepatic cyst causing obstructive jaundice was first reported in 1950 by Carabati et al [1], and to date, over 50 cases have been reported [5]. The treatment of such cysts includes percutaneous drainage, fenestration, and liver resection by laparotomy or laparoscopy. In the present case, we selected non-surgical treatment for initial management of the infected cyst in consideration of the patient’s quality of life, after which bile duct obstruction was relieved and her jaundice subsided. If ablation is not performed, a cyst will usually recur after temporary percutaneous drainage without injection of a sclerosant. It has been suggested that the injection of a sclerosant is effective because it kills the endothelial cells that line the cyst wall. For sclerotherapy of hepatic cysts, ethanol [6], minocycline hydrochloride [7], monoethanolamine oleate, and Pantopaque [8] have been used. The prognosis of solitary non-parasitic hepatic cysts associated with jaundice is generally good [5]. Recurrence of infected hepatic cysts is not common after drainage, possibly because the secretory lining cells are devitalized by infection.

It is rare for compression of the bile duct by neighboring vessels to cause obstructive jaundice, and the reported cases of arterial bile duct compression have involved the right hepatic artery [9-11], a hepatic artery aneurysm [12], and an aberrant celiac artery [13]. It has also been reported that the venous system, such as distended collateral veins in patients with portal hypertension, can also cause bile duct compression [14]. Kumada et al. advocated the term right hepatic artery syndrome for compression of the common hepatic duct by the right hepatic artery leading to obstructive jaundice [10]. Aging has been suspected to be associated with this condition, possibly because compression of the bile duct by the right hepatic artery may result from changes in the vessel itself, such as arteriosclerosis, or may occur because changes of a nearby organ influence the artery. Watanabe et al. and Kumada et al. have reported patients who had intrahepatic stones associated with bile duct compression by the right hepatic artery [9, 10]. In their patients, arterial compression of the bile duct might have led to stasis of bile and subsequent development of intrahepatic stones.

In our patient, there was a band-like filling defect of the hilar bile duct on ERC, and encasement of the right hepatic artery was seen at the same level on abdominal angiography. In addition, cholangioscopy revealed stenosis and mucosal erosions of the bile duct. It seems that these inflammatory changes and the band-like filling defect of the bile duct on ERC were due to compression by the right hepatic artery due to displacement by the hepatic cyst.

In conclusion, we reported a rare case of obstructive jaundice associated with a hepatic cyst and encasement of the right hepatic artery. When a band-like filling defect of the common hepatic duct is observed near a large liver cyst, right hepatic artery syndrome should be taken into consideration.
